# A QoS-Guaranteed Coverage Precedence Routing Algorithm for Wireless Sensor Networks

**DOI:** 10.3390/s110403418

**Published:** 2011-03-24

**Authors:** Joe-Air Jiang, Tzu-Shiang Lin, Cheng-Long Chuang, Chia-Pang Chen, Chin-Hong Sun, Jehn-Yih Juang, Jiun-Chuan Lin, Wei-Wen Liang

**Affiliations:** 1 Department of Bio-Industrial Mechatronics Engineering, National Taiwan University, No. 1, Sec. 4, Roosevelt Road, Taipei 106, Taiwan; E-Mails: d98631001@ntu.edu.tw (T.-S.L.); richardchuang@ntu.edu.tw (C.-L.C.); supercjb@pie.com.tw (C.-P.C.); 2 Department of Geography, National Taiwan University, No. 1, Sec. 4, Roosevelt Road, Taipei 106, Taiwan; E-Mails: chsun@tgic.org.tw (C.-H.S.); jjuang@ntu.edu.tw (J.-Y.J.); jclin@ntu.edu.tw (J.-C.L.); 3 Taiwan Geographic Information System Center, 6F 7 Roosevelt Road, Sec. 1, Taipei 10092, Taiwan; E-Mail: samuel_liang@tgic.org.tw (W.-W.L.)

**Keywords:** quality of service (QoS), routing algorithm, sensing coverage problem, wireless sensor network (WSN)

## Abstract

For mission-critical applications of wireless sensor networks (WSNs) involving extensive battlefield surveillance, medical healthcare, *etc.*, it is crucial to have low-power, new protocols, methodologies and structures for transferring data and information in a network with full sensing coverage capability for an extended working period. The upmost mission is to ensure that the network is fully functional providing reliable transmission of the sensed data without the risk of data loss. WSNs have been applied to various types of mission-critical applications. Coverage preservation is one of the most essential functions to guarantee quality of service (QoS) in WSNs. However, a tradeoff exists between sensing coverage and network lifetime due to the limited energy supplies of sensor nodes. In this study, we propose a routing protocol to accommodate both energy-balance and coverage-preservation for sensor nodes in WSNs. The energy consumption for radio transmissions and the residual energy over the network are taken into account when the proposed protocol determines an energy-efficient route for a packet. The simulation results demonstrate that the proposed protocol is able to increase the duration of the on-duty network and provide up to 98.3% and 85.7% of extra service time with 100% sensing coverage ratio comparing with LEACH and the LEACH-Coverage-U protocols, respectively.

## 1. Introduction

When applying a sensor network to fields that involve emergency events, such as battlefield surveillance, medical healthcare, illegal smuggling, *etc.*, the primary concern is to preserve all valuable data acquired from the targeted area without any losses. For instance, sensor nodes have been deployed in the military area, and each sensor node was equipped with a sound sensor. The sound sensors can detect the sound made by the soldiers and military vehicles in a limited sensing range. The sensor network needs to discover any unusual sound made by enemies; hence, it is crucial to have low-power, new protocols, methodologies and structures for transferring data and information in a network with full sensing coverage capability for an extended working period.

In recent years, the goal of constructing wireless sensor networks (WSNs) provides an *ad hoc* communication model serving in a specific region with mission-critical applications. WSNs consist of a great number of sensor nodes with wireless communication capability. With the advantage of integrated circuits and wireless communication technology, wireless sensor nodes have been manufactured using low-cost and low-power design for practical applications [1,2]. Due to the limited energy resources of sensor nodes, many previous studies, such as routing algorithms, coverage control, power management, node localization, and medium-access control, have been proposed to deal with the limited energy issue [3]. In many applications, WSNs are organized as clusters, which have been widely studied in recent years. The clustered architecture decreases the opportunity of communication overhearing and power dissipation of sensor nodes. The clustered architecture groups up sensor nodes that are nearby. The sensed data is sent to a cluster head for data fusion and aggregation. Thus, the size of the sensed data sent to the sink can be reduced, and the energy consumption of sensor nodes is further reduced. The clustered architecture has been proven to be successful in saving energy and prolonging the network lifetime [4–6]. In addition to energy efficiency, it is critical to maintain sensing coverage over the entire targeted area. The coverage preservation is a basic requirement for fulfilling the quality of service (QoS) in many mission-critical applications, such as battlefield or border surveillance [7]. Any hole that occurs in the coverage of a given network might be fatal and not be tolerable [8].

The network designed for mission-critical applications using WSN technologies exploits the features of *ad hoc* networking. The primary goal of the mission-critical network is to prevent the sensed data from being routed through sparsely populated areas covered by a small number of sensor nodes [9]. The idea behind this approach is that the nodes in the sparsely populated areas are less used as packet routers. Thus, these nodes can utilize their energy resources to collect data for a longer working period. Furthermore, in a mission-critical application, dynamic deployment of sensor nodes for the rapid exploration of the emergency area is essential. The sensor nodes should be able to be rapidly deployed without concerning the network topology that influences the sustenance of full sensing coverage.

In this study, we integrate energy-efficiency and coverage-preserving techniques in a cooperative manner. A novel energy-aware coverage-preserving hierarchical routing protocol (referred as ECHR) is presented to maximize the working time of full coverage in a given WSN regardless of the deployment patterns of the sensor nodes. The basic idea of the proposed ECHR algorithm is to take the remaining energy of the nodes as well as the coverage redundancy of its sensing ranges into consideration when selecting a root node. Intuitively, the sensor nodes deployed in a densely populated area have a higher probability to be selected as the root node in each round. These nodes are frequently chosen to be the root node in the early stage of sensing phase, because the loss of nodes in the densely populated area is not significant for the network coverage. In addition, an energy-aware hierarchy routing mechanism is also proposed to determine an energy-efficient route when transmitting a packet that contains the sensed data.

The remainder of this paper is organized as follows. Section 2 provides the-state-of-the-art review on related works. Section 3 explains definitions of the radio transmission model and the coverage model of the WSNs. The proposed ECHR protocol is presented in Section 4. Section 5 demonstrates the simulation results yielded by the proposed ECHR protocol. Finally, concluding remarks are given in Section 6.

## 2. Related Works

Due to limited energy and communication ability of wireless sensor nodes, a number of energy-efficient routing protocols have been proposed to prolong their lifetime [10]. Some approaches select cluster heads according to residual energy of sensor nodes [11,12]. Others transmit data packets by finding the shortest or the most reliable path between any paired nodes [13–15]. A detailed review of energy-efficient protocols is given as follows.

The low-energy adaptive cluster hierarchy (LEACH) [4] is one of the most well-known routing protocols to date. LEACH chooses cluster heads in a network to collect the data transmitted by remote sensor nodes. With data fusion and aggregation functions, the cluster heads are able to combine and compress the sensed data into significantly smaller-sized packets. Since the sensed data is fused in each hop, the energy consumption caused by radio transmission can be greatly reduced. Handy *et al.* modified the cluster-head selection algorithm originated from the LEACH protocol to reduce the overall energy consumption of the network [11]. The algorithm takes the residual energy of nodes into account when selecting a proper cluster-head, and also improves the energy-balancing of the network that contributes to prolong the network lifetime. In [12], they further utilize node proximity that allows sensor nodes to join the closest cluster-head in order to minimize the communication cost inside the cluster. However, in [4,11,12], the cluster heads transmit sensed data to a base station (BS) directly, and the long distance transmissions consume greater energy.

By transmitting the sensed data to the BS using a multi-hop mechanism, the energy consumption of each sensor node can be further reduced. Another protocol focusing on energy-efficiency, the energy-efficient unequal clustering (EEUC) protocol [16], used a multi-hop transmission mechanism to connect cluster heads and a BS. It utilized an unequal probability density to select cluster heads to reduce the loading of nodes near the BS. The power efficient gathering algorithm in sensor information systems (PEGASIS) [17], also emphasizing the idea of reducing energy consumption, allows the sensor nodes to have communication capability to transmit the sensed data to the BS. The PEGASIS minimized the energy consumption of sensor nodes by selecting only one cluster-head in each round. Both EEUC and PEGASIS protocols have been found to outperform the LEACH protocol.

The focus of these famous algorithms is to reduce overall energy consumption of the network, so the network lifetime can be extended. However, the full sensing coverage at any given time during a sensing phase is not guaranteed, unless the sensor nodes are equally distributed. In many practical applications, sensor nodes are not equally distributed over the monitoring area, and thus the sensing coverage is hard to maintain under such a circumstance [8]. This drawback would cause a great number of coverage holes, which makes the algorithms unsuitable for mission-critical applications.

The coverage-time concept was proposed in [15], where the energy-balancing is taken into consideration in intra- and inter-cluster communications. In this protocol, the cluster-head chooses the shortest hop-count path for the data transmission in inter-cluster, and the sizes of all clusters are the same for energy-balancing. Moreover, Tsai [18] presented a coverage-preserving routing protocol, named “LEACH-Coverage-U”. In contrast with the aforementioned protocols, the LEACH-Coverage-U protocol calculated the overlap sensing areas of all sensor nodes and then selected cluster heads starting from the nodes in a highly overlapped area. The simulation results showed that the LEACH-Coverage-U protocol could prolong the network lifetime compared with existing protocols. Moreover, the coverage and connectivity aware routing protocol based on neural networks was proposed in [19]. The cluster-head selection and optimized route of data transmission using adaptive learning in neural networks could cause a huge computation burden for sensor nodes. Moreover, Noh *et al.* [20] proposed a Coverage-Preserving Scheme (BCoPS), which is a novel approach that allows a BS to maintain the network with consideration of various factors, such as network coverage, wake-up strategies, and cluster formation. Although the proposed works in [18–20] prolong network lifetime, they cannot guarantee full sensing coverage of the network. Retaining full sensing coverage is an important issue when losing any sensed data is not affordable.

The full coverage issue was mentioned in [21], and the authors proposed several cost metrics (coverage and energy-aware costs) for different application scenarios. The sensor nodes deployed in a densely populated area can serve as cluster heads, active sensor nodes, and routers. The cost metrics are not only used for the cluster-head selection but also for active node selection and routing-table update. However, using these metrics causes a larger computational burden on sensor nodes. Wang *et al.* [22] presented the coverage-aware clustering protocol (CACP) for randomly deployed networks, which simplifies the cost metric for cluster-head selection and active node selection. The CACP outperforms the protocol proposed in [21]; however, each cluster-head consumes much energy when the cluster head directly transmits the aggregated data to the BS.

## 3. Problem Formulation

As mentioned above, the purpose of this study is to design a coverage precedence routing algorithm for mission-critical applications. The primary goals of a mission-critical network is to prevent the sensed data from being routed through sparsely populated areas covered by a small number of sensor nodes, and to maximize the network lifetime under full coverage. To accomplish these goals in the cluster-based WSNs, the problem here can be formulated as a root selection problem. Generally, the *sink node* (or known as *base station*) is assumed to be deployed at any location inside or outside of the monitoring area, and a root node is chosen to collect all sensed data and then transmit it to the base station. According to the general radio transmission model, transmitting a packet through a long path consumes great energy. The idea to achieve these tasks is that the nodes in the sparsely populated areas are less chosen to be root nodes (or as packet routers). Before we introduce the proposed algorithm, the mathematical models for network and coverage are defined in the following subsections.

### 3.1. Network Configuration

**Figure 1.**
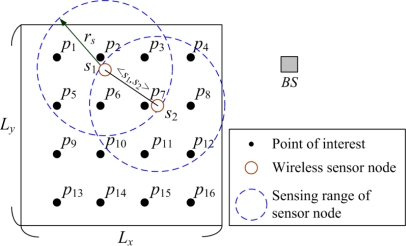
Example for the coverage model of sensor node.

Suppose a WSN is a hybrid network with a BS having additional processing power and *n* remote sensor nodes deployed in an *L_x_* × *L_y_* monitoring area. There are *m* points of interest (abbreviated as POI) in the monitoring area. The location of the sensor node is assumed to be known a priori. Thus, the network is represented by the Euclidean graph *G*, and *G* = (*V*, *E*), as depicted in Figure 1, with the following properties:

➢ *V* is a set of nodes in the network and *V* = {*S*, *BS*}, where *S* is a set of sensor nodes with a circular sensing range *r_s_* and *S* = {*s*_1_, *s*_2_, …,*s_n_*}, *BS* is the base station, and *n* is the number of sensor nodes.
➢ Sensor nodes in *V* of the network know their location information.
➢ <*s_i_*, *s_j_*> ∈ *E*, where *s_i_* ≠ *s_j_*. It is sustainable if the distance between *s_i_* and *s_j_* is shorter than the communication range of the sensor nodes in *V*.
➢ All sensor nodes in *S* are homogeneous, *i.e.*, their sensing range, wireless communication capability, and initial power are identical.
➢ All nodes in *V* are stationary after the deployment.
➢ All nodes in *V* have the power management capability; their radio power can be dynamically adjusted according to the transmission distance.
➢ *BS* can be deployed at any location inside or outside of the monitoring area.

### 3.2. Coverage-Aware Cost Metric

As mentioned in the Section 1, the primary goal of the proposed ECHR algorithm is to prevent the sensed data from being routed through sparsely deployed areas. Therefore, the nodes in these areas can be less used as data routers but more used as data collectors. This coverage-preservation task requires a coverage-aware cost metric to calculate the overall coverage ratio, the distribution of the remaining energy, and the overlapped sensing area covered by the neighboring sensor nodes.

We assume that the mission-critical application requires every part of the area to be covered by the sensor nodes in *V*. Each sensor node performs a sensing task on the points of interest (POI) located within its sensing area. The sensing area of each node is approximated by a circular area around the node with radius *r_s_*. Such a model is the simplest and the most common method in determining the sensing coverage of a given WSN. A set of POIs that will be monitored is denoted by *P*, where *P* = {*p_j_*, *j* = 1, …, *m*}. If the distance between a sensor node *s_i_* and a POI *p_j_* is shorter than, or equal to, *r_s_*, the coverage set of the sensor node *s_i_* is then defined by:

(1) $$C\left(s_{i}\right)=\left\{p_{j}|d\left(s_{i},p_{j}\right)\leq r_{s}\right\},$$

where *d*(*s_i_*, *p_j_*) is the Euclidean distance between the node *s_i_* and a POI *p_j_*. For example, the set of POIs covered by the sensor node *s*_1_ in Figure 1 is *C*(*s*_1_), and *C*(*s*_1_) = {*p*_1_, *p*_2_, *p*_3_, *p*_5_, *p*_6_, *p*_7_}. Usually, multiple sensor nodes in the network cover the same POI. This case is called the coverage redundancy. According to the definition given above, the subset of POIs that are simultaneously covered by multiple sensor nodes can be determined by:

(2) $$O\left(s_{i}\right)=C\left(s_{i}\right)\cap \left(C\left(s_{1}\right)\cup C\left(s_{2}\right)\cup ...\cup C\left(s_{i-1}\right)\cup C\left(s_{i+1}\right)\cup ...\cup C\left(s_{n}\right)\right),$$

where *O*(*s_i_*) is the intersection of the sets of POI covered by *s_i_* and other sensor nodes. If *O*(*s_i_*) = *C*(*s_i_*), the sensor node *s_i_* is identified as a redundant node. For a given WSN, the coverage ratio *R* of a given WSN is thereby defined by:

(3) $$R=\frac{\left\|\overset{n}{\underset{i=1}{\cup }}C\left(s_{i}\right)\right\|}{\left\|P\right\|}\times 100\%=\frac{\left\|C\left(s_{1}\right)\cup C\left(s_{2}\right)\cup ...\cup C\left(s_{n-1}\right)\cup C\left(s_{n}\right)\right\|}{\left\|P\right\|}\times 100\%\;,$$

where ‖*P*‖ is the number of POIs in *P*. In addition, if a sensor node *s_i_* runs out of its energy, *C*(*s_i_*) in Equation (1) is deflated to an empty set.

## 4. The Proposed ECHR Algorithm

The focus of this study is to apply a WSN to mission-critical applications. Extending network lifetime without the risk of data loss is the basic QoS requirement in such applications. In order to prolong the working time of the network with a full coverage of *R*, *i.e.*, *R* = 100%, a root node selection mechanism based on energy-balancing and coverage-preserving techniques is presented. An energy-aware hierarchical routing algorithm is proposed to determine an energy-efficient path to route the data packets to the *BS*. In each round, the selection of the root node is decided by the *BS*, and energy-aware hierarchical routing algorithm is applied to each node. Detailed descriptions are provided in the following subsections.

### 4.1. Selection of the Root Node

In each round of performing the ECHR protocol, the first step is to select the root node. Generally, the *BS* is assumed to be deployed at any location inside or outside of the monitoring area. According to the radio transmission model described in Section 3, transmitting a packet through a long path consumes greater energy. Since high energy consumption is not suitable for a power-limited network, the root node selection method is essential. In each round, we compute the root node weight of each node *n_i_* by:

(4) $$\alpha_{i}={\left(q_{i}\right)}^{\tau_{1}}\times {\left(\frac{\left\|O\left(s_{i}\right)\right\|}{\left\|C\left(s_{i}\right)\right\|}\right)}^{\tau_{2}}\times \left(\frac{1}{d(s_{i},BS)}\right),$$

where *q_i_* is the residual energy of *s_i_*, *d*(*s_i_*, *BS*) is the Euclidean distance between node *s_i_* and the *BS*, and *τ*_1_ and *τ*_2_ are the weighting coefficients for the residual energy factor and the coverage factor, respectively. After the weights of all nodes are computed, we can form a set of root node weights ***α*** by:

(5) $$\text{α}=\left[\begin{array}{c} \alpha_{1}\\ \alpha_{2}\\ \vdots \\ \alpha_{n} \end{array}\right].$$

Next, we can select the *H*-th node of the network to be the root node via:

(6) $$H=\text{arg max}\;\text{α}=\underset{i\in S}{\text{arg max}}\;\alpha_{i}\;,$$

where *S* is a set of sensor nodes in the network.

In each round, the root node broadcasts a beacon message with a packet format that includes its ID, residual energy, and level, toward other sensor nodes. Nodes that receive the beacon message of the root node are called the first level nodes. The first level nodes broadcast the beacon message, and the nodes that receive the beacon message from first level nodes are called the second level nodes. With the hierarchical broadcasting, each node is able to establish its level and receive the information of the neighboring nodes. After all sensor nodes broadcast the beacon message, each node is able to establish the neighbor set of its neighboring nodes.

### 4.2. Energy-Aware Hierarchical Routing Algorithm

Like the multi-hop transmission mechanism mentioned earlier, the communication range *C_r_* of any sensor node in network can be dynamically adjusted to reduce the power dissipation in data transmission. By shrinking the communication range *C_r_*, the sensor nodes are not able to transmit the sensed data to distanced nodes. Hence, all sensed data will be routed to the neighboring nodes of the sensing node by the multi-hop mechanism.

Due to the hop-count information provided in the beacon message, the sensor nodes are able to determine which neighboring nodes are closer to the root node. The closest neighboring node can be chosen as the parent node to relay the sensed data. In each round, each sensor node transmits a sensed data to the root node. First, we compute the path weight for transmitting the data from a source node *s_i_* to a destination node (or relay node) *s_p_* by:

(7) $$\beta_{i,p}={\left(\frac{1}{d\left(s_{i},s_{p}\right)}\right)}^{\lambda_{1}}\times {\left(q_{p}\right)}^{\lambda_{2}},$$

where *d*(*s_i_*, *s_p_*) is the Euclidean distance between nodes *s_i_* and *s_p_*, and *λ*_1_ and *λ*_2_ are weight coefficients to adjust the relative importance of the distance factor and the residual energy factor, respectively. After the values of all *β_i,p_* are determined, we can organize them in a set of weights ***β**_i_* by:

(8) $${\text{β}}_{i}=\left[\begin{array}{c} \beta_{i,\;1}\\ \beta_{i,\;2}\\ \vdots \\ \beta_{i,\left\|N(i)\right\|} \end{array}\right].$$

where ‖*N*(*i*)‖ is the number of parent candidates of node *s_i_*. Subsequently, we can select the *G_i_*-th node to be the parent node for data transportation by:

(9) $$G_{i}=\;\text{argmax}\beta_{i}=\;\underset{p\in N(i)}{\text{arg max}}\beta_{i,p}\;,$$

where *N*(*i*) is the set of parent candidates of node *s_i_*. Hence, the sensor node *s_i_* is likely to choose a closer node with greater residual energy. The task of data relaying that requires high energy consumption can be then assigned to a possible neighboring node without creating any hot-spot in the network. After all of the sensed data are collected by the root node, the root node aggregates the data, and then transmits it to the *BS*.

**Figure 2.**
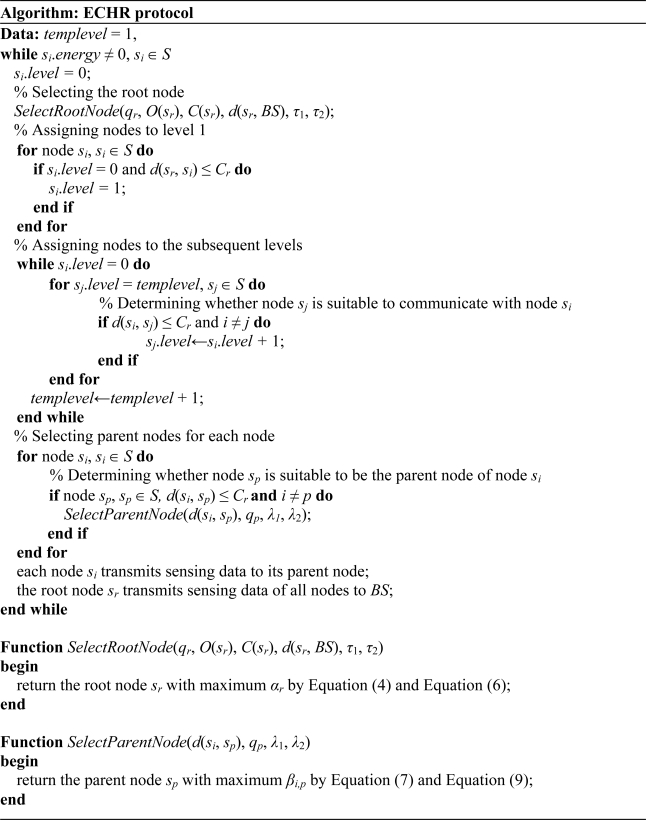
Pseudo code of the proposed ECHR protocol.

**Figure 3.**
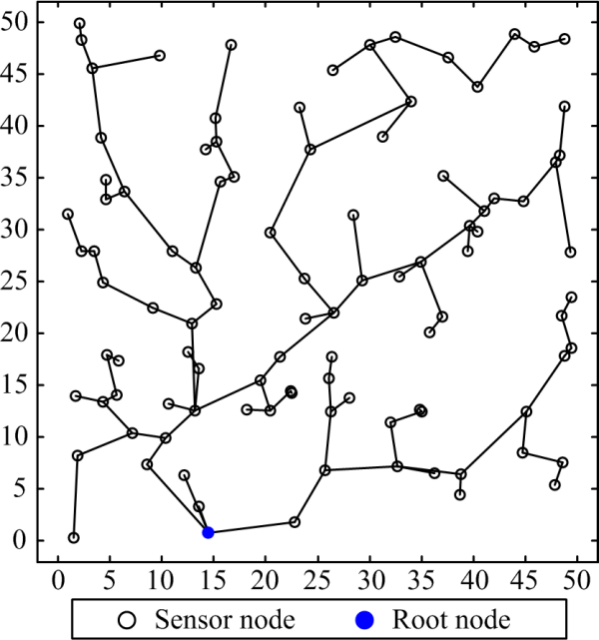
Data transmission paths using the ECHR algorithm for a specific network topology.

The ECHR algorithm utilizes the multi-hop transmission mechanism as a spanning tree topology to reduce the power dissipations in the packet transmission phase. The pseudo code of the proposed ECHR algorithm is shown in Figure 2. In the ECHR algorithm, *s_i_*.*energy* is the residual energy of node *s_i_*, and *s_i_*.*level* is the number of hops when transmits data to the root node. Figure 3 shows the data transmission paths of nodes using the ECHR algorithm for a specific network topology.

## 5. Simulations

### 5.1. Radio Transmission Model

In this study, we adopt the radio model stated in [4] to calculate the energy consumed by radio transmissions. There are two primary factors that involve in the radio model: *E_elec_* and *ɛ_amp_*. *E_elec_* represents the energy consumption per bit by either the electrical circuits of the transmitter node *s_t_* or the receiver node *s_r_* in *S*, and *ɛ_amp_* is the energy consumption per bit by the signal amplifier of the transmitter node *s_t_*. The radio model is formulated by:

(10) $$E_{\mathrm{Tx}}(k,d)=k(E_{\mathrm{elec}}+{ɛ}_{\mathrm{amp}}d^{\gamma })$$

(11) $$E_{\mathrm{Rx}}(k)={\mathrm{kE}}_{\mathrm{elec}},$$

where *E_Tx_* is the energy consumption for transmitting data, *E_Rx_* denotes the energy consumption by receiving data, *d* is the distance between the transmitter node *s_t_* and the receiver node *s_r_*, and *γ* is the path loss exponent.

For the nodes that serve as intermediate nodes, besides energy consumption of transmitting data and receiving data, extra energy consumption is required to complete the tasks of aggregating and compressing the sensed data. When an intermediate node receives a packet of length *k*-bits, the energy consumption can be formulated by *E_Rx_*. The packet is then compressed into a packet of *μ* × *k* bits, and the energy consumption can be formulated by *k* × *E_DA_*, where *E_DA_* is the energy consumption per bit in data aggregation, and *μ* is the compression coefficient. After the data aggregation, the node transmits the aggregated data to the next-hop node. Hence, the total energy consumption of an intermediate node for receiving and transmitting a packet, denoted by *E_int_*, is:

(12) $$E_{\mathrm{int}}=E_{\mathrm{Rx}}+{\mathrm{kE}}_{\mathrm{DA}}+{\mu E}_{\mathrm{Tx}}.$$

It is worth noting that this radio transmission model may not be consistent with the specification of some radio-frequency transceivers. However, this radio transmission model has been widely utilized in many previously proposed studies. In order to conduct a fair comparison between the performances of the proposed approach and other existing methods, as well as to focus the scope of this study on the routing issue, the aforementioned radio transmission model is utilized.

### 5.2. Network Parameters

The proposed method is most suitable for circumstances in which losing any sensing data is not acceptable, e.g., WSN-based perimeter surveillance in battlefields or other high security areas [23]. According to this scenario, sensor nodes can be randomly deployed by helicopters or aircrafts [7]. They are then self-organized into a functional WSN for comprehensive and continuous perimeter surveillance. In addition, the proposed method is also suitable for the WSN-based smart healthcare that requires continuous and remote monitoring [24]. Here, we take the battlefield surveillance as an example; a network with 100 sensor nodes is randomly distributed over a battle area of 50 × 50 m^2^. The deployed nodes equipped with vibration sensors that can detect any object passing through the area within its sensing range. The sensing range *r* is set at 7.5 m. In addition, this battlefield consists of 2,500 POIs that are grid distributed. In each round live nodes need to report the sensed data to the *BS*, and the *BS* is located at the coordinate (25, −50). In order to prevent long distance transmissions and to reduce power dissipation in data transmission, a suitable communication range *C_r_* is chosen to make the average hop-count of nodes equal to 7.5. The initial energy of all nodes is assumed to be 1 joule, and the nodes cannot be recharged. Furthermore, the parameters of the radio model are summarized in Table 1, which are the same as those adopted by [18] in order to perform fair comparisons between the proposed method and the previous studies. The path loss of the radio model is set the same as the previous studies [11,18], in which *γ* is 2 for data transmissions between nodes in a free-space. We specifically set *γ* to 2.5 for the long range data transmissions from the root node to the BS [25]. The compression coefficient *μ* of Equation (12) is set at 0.05. In this study, each node should have some specific data which is different than other nodes, and the setting of compression coefficient *μ* set at 0.05 is much suitable in the real-world works. Hence, the compression coefficient *μ* is set at 0.05 in this work to evaluate the ECHR performance. Simulation results are obtained by averaging those obtained from 100 network topologies.

In this section, the performance analysis of the proposed ECHR protocol is conducted via MATLAB simulation. The simulation consists of two parts. In the first part, the performance of the proposed ECHR is evaluated using different weighting coefficients. In the second part of the simulation, the performance of the ECHR protocol is compared with those of the LEACH and the LEACH-Coverage-U via numerical simulation.

### 5.3. Performance Evaluation of the ECHR Protocol under Varying Weighting Coefficients

The main goal of this simulation is to evaluate the performance of the ECHR protocol when applying it to a network with different weighting factors. The lifetime of the network with a full sensing coverage ratio achieved by using the ECHR protocol is also investigated. These factors are used to select a root node in each round and determine an energy-efficient route for each node. The framework of the divide and conquer method [26] is borrowed to dissect the proposed ECHR protocol. The first part of simulation is to find optimal weighting coefficients of Equation (4), *τ*_1_ and *τ*_2_, in the root node selection mechanism. In the second part of simulation, the effects of the parameters of Equation (7) are identified in the energy-aware hierarchical routing mechanism, *λ*_1_ and *λ*_2_.

**Figure 4.**
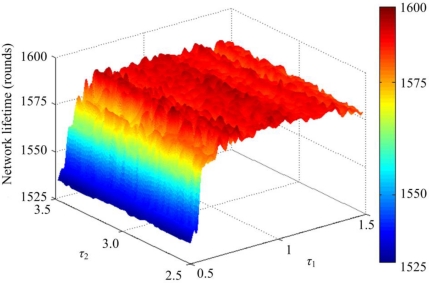
Plot of network lifetime with 100% sensing coverage *versus τ*_1_ and *τ*_2_ when *λ*_1_ = 0.7 and *λ*_2_ = 3.3.

In the first part of simulation, a same network model is setup as mentioned above. The network with 100 nodes is deployed in an area of 50 × 50 m^2^. In order to study the effect of weighting coefficients on the residual energy factor and the coverage factor in Equation (4), *λ*_1_ is set at 0.7 while *λ*_2_ is set at 3.3. Figure 4 shows three-dimensional plots of the lifetime of the network with a 100% sensing coverage ratio *versus τ*_1_ and *τ*_2_. After examining the simulation results, it is found that the optimum value of network lifetime with a 100% sensing coverage ratio can be obtained when *τ*_1_ = 1 and *τ*_2_ = 3.1.

**Figure 5.**
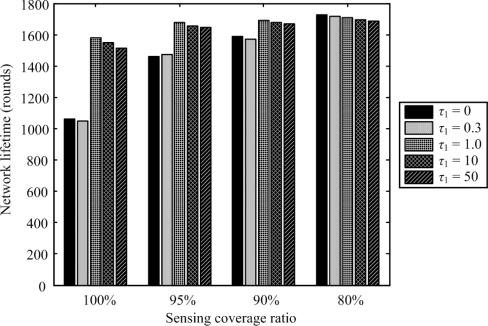
Comparison of network lifetimes under different sensing coverage ratios with varying *τ*_1_ when set *τ*_2_ = 3.1.

Figure 5 shows the coverage-lifetime comparison under varying *τ*_1_ when set *τ*_2_ is set at 3.1. The coverage-lifetime demonstrated in Figure 5 is the network lifetime when 100%, 95%, 90%, and 80% of network coverage is preserved, respectively. When *τ*_1_ = 1.0, the network lifetime can provide a full sensing coverage over other *τ*_1_ values. In this case, if the root node selection does not take the residue energy into account (*i.e.*, *τ*_1_ = 0), the redundant nodes will always be chosen as the root node and their energy will run out fast. Under this circumstance, however, the data collected by active nodes needs to be transmitted for a long distance, because fewer intermediate nodes are used to transmit data. As a result, the active nodes run out of energy more quickly. On the contrary, the proposed root selection mechanism puts an emphasis more on energy-balancing than the coverage factor does when *τ*_1_ is set at a large value, say 50. Such a situation leads to a scenario that the redundant node might not be chosen as the root node, network lifetime with a full sensing coverage may not last longer.

**Figure 6.**
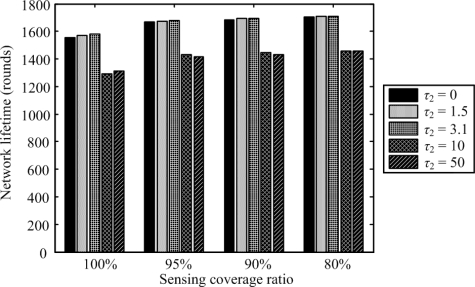
Comparison of network lifetimes under different sensing coverage ratios with varying *τ*_2_ when set *τ*_1_ = 1.

Figure 6 shows the coverage-lifetime comparison results under different values of *τ*_2_ when setting *τ*_1_ at 1.0. When the sensing coverage is 100%, the network lifetime is the longest, if *τ*_2_ = 3.1. In this case, the coverage factor in the root node selection is not taken into account (*i.e.*, *τ*_2_ = 0). Such a situation is similar to the scenarios where *τ*_1_ is set at a large value, as mentioned above. Moreover, the impact of the coverage factor on the root node selection mechanism is greater than that of the residual energy factor if *τ*_2_ is set at a large value, e.g., 50. In this circumstance, the performance of the network is similar to the case where *τ*_1_ = 0.

**Figure 7.**
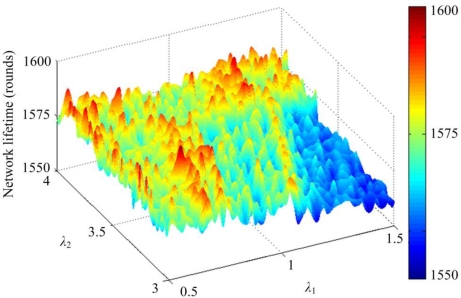
Plot of network lifetime with 100% sensing coverage *versus λ*_1_ and *λ*_2_ when *τ*_1_ = 1 and *τ*_2_ = 3.1.

In addition, we analyze the effects of varying weighting coefficients of the distance factor, *λ*_1_, and the residual energy factor, *λ*_2_, of Equation (7) on the energy-aware hierarchical routing algorithm. Figure 7 shows three-dimensional plots of the network lifetime with a 100% sensing coverage *versus λ*_1_ and *λ*_2_, when *τ*_1_ = 1 and *τ*_2_ = 3.1. After an extensive series of simulations, the optimal value is located at the area where 0.5 ≤ *λ*_1_ ≤ 1.5 and 3.0 ≤ *λ*_2_ ≤ 4.0. Here, we find optimum weighting coefficients, *λ*_1_, equal to 0.7, and *λ*_2_, equal to 3.3, with the maximum network lifetime under a 100% sensing coverage ratio.

Table 2 depicts the coverage-lifetime comparison under varying *λ*_1_ when *λ*_2_ is set at 3.3. The network in which *λ*_1_ = 0.7 provides longer coverage-lifetime under a 100% sensing coverage ratio, compared to other values of *λ*_1_. For instance, when *λ*_1_ = 0, the improvement in the network coverage-lifetime reaches 43%. In this case, the distance factor of the energy-aware hierarchical routing algorithm is not taken into account which means that farther neighboring nodes with higher residual energy can be chosen as the parent node. Under this situation, the energy consumption in data transmission increases. However, this fact causes the network to fast run out of power. On the other hand, the impact of the distance factor of Equation (7) on the energy-aware hierarchical routing mechanism is greater than that of the residual energy factor when *λ*_1_ is set at a large value, say 50. Such a situation drives the node to choose the nearest node as a parent node which could be a critical node. If the essential node over used as an intermediate node, the node may quickly run out of energy, and thus causing the network cease working.

Table 2 also shows the results of coverage-lifetime comparison using different values of *λ*_2_ when *λ*_1_ = 0.7. The ECHR protocol has the best performance when *λ*_2_ = 3.3, despite the similarity in the coverage-lifetime under a 100% coverage ratio. If the residual energy factor of Equation (7) is not taken into account (*i.e.*, *λ*_2_ = 0), and the distance factor, *λ*_1_, will be the only factor that influences the result of selecting parent node. This case is similar to the scenario that *λ*_1_ is set at a large value, as mentioned above. Furthermore, the effect of the residual energy factor is greater than that of the distance factor when *λ*_2_ is set at a large value, e.g., 50. This means that a node will choose its parent node depending on which one of possible parent candidates has the highest residual energy. Because the distance factor is also considered in this case, the performance of the network where *λ*_1_ = 0.7 and *λ*_2_ = 50 is better than that of the network where *λ*_1_ = 0 and *λ*_2_ = 3.3.

### 5.4. Performance of the ECHR Protocol

In this section, the performance of the ECHR protocol is compared with those of the LEACH [4] and the LEACH-Coverage-U [18] protocols via an extensive series of simulations. The simulations using different protocols are ceased once all nodes run out of energy, and the comparison results generated. In the case I, the same network model in both approaches [4,18] mentioned above is used to examine the protocols. The LEACH and the LEACH-Coverage-U both set their *BS* in a remote place, and each node can directly transmit data to the *BS*. Such a condition, however, is not suitable for a real-world environment, because each tiny low-cost sensor node does not have such strong communication capability. Consequently, in the simulation a gateway is located in the center of the monitoring area, *i.e.*, located at (25, 25), equipped with a long distance wireless communication module (e.g., the global system for mobile communications module) capable of transmitting the sensed data to the *BS*. In each round, sensor nodes transmit sensing data to the gateway, and the gateway sends the sensing data of sensor nodes to the *BS*. Moreover, the path loss exponent *γ* of Equation (10) is set at 2.5 when transmitting data from the root node to the gateway [4,18]. In the case II, the gateway is served as the *BS* and located in the center of the monitoring area. Simulation results are presented as follows.

**Figure 8.**
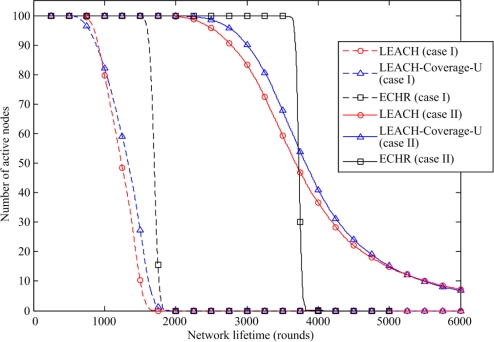
Comparison of the active nodes of the proposed ECHR protocol with those of other protocols.

Figure 8 shows the number of active sensor nodes *versus* the simulation rounds. In the case I, the LEACH and the LEACH-Coverage-U protocols lose their first node around the 600^th^ round. The proposed ECHR protocol is able to maintain all sensor nodes alive till the 1,500^th^ round, which is approximately 2.5 times longer than those generated by the LEACH and the LEACH-Coverage-U protocols. Moreover, the lifetime of first node that runs out of its energy using the proposed ECHR protocol is longer than those using the LEACH and the LEACH-Coverage-U protocols in the case II. By contract, using the ECHR protocol, after the first node runs out of energy, the number of the active nodes sharply falls. This is because the proposed ECHR protocol is able to equalize the energy consumption over the entire network. Furthermore, by maintaining nodes surviving longer time when relaying data, the energy consumed in transmission can be significantly reduced. Hence, using the proposed ECHR protocol guarantees the WSN with high coverage precedence when applying the WSN to specific mission-critical areas, such as military surveillance and e-health care.

**Figure 9.**
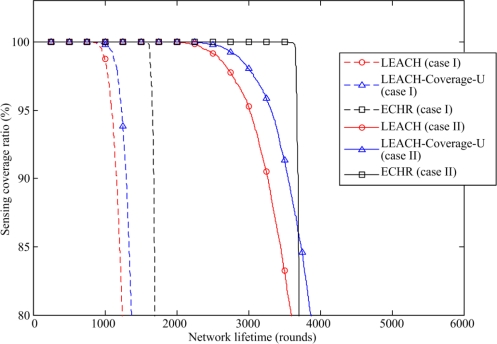
Comparison of the coverage ratio of the proposed ECHR protocol with those of other protocols.

Figure 9 depicts the coverage ratio *versus* the simulation rounds. The proposed ECHR protocol performs relatively well when comparing to the LEACH and the LEACH-Coverage-U protocols. In the case I, for example the ECHR protocol maintains 100% coverage ratio until the 1,590^th^ round, but the coverage ratios of LEACH protocol and the LEACH-Coverage-U protocol drop from 100% at the 802^th^ and the 856^th^ round, respectively. In other words, compared to the LEACH and the LEACH-Coverage-U protocols, the proposed ECHR protocol provides 98.3% and 85.7% increase in service time with a 100% sensing coverage ratio. The ECHR protocol also outperforms other protocols in the case II, and its network lifetime can be extended to nearly 1,250 rounds.

**Figure 10.**
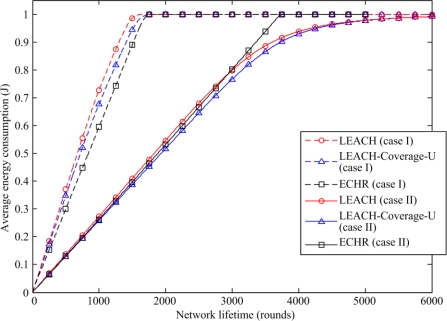
Comparison of the average energy consumption of the proposed ECHR protocol with those of other protocols.

Figure 10 depicts the average energy consumption of each node *versus* the simulation rounds when using three different protocols in the cases I and II. The average energy consumption of the ECHR protocol steadily increases during the simulation due to its energy-balancing capability. Moreover, the comparison between the results yielded by the LEACH and the LEACH-Coverage-U protocols clearly indicates that the 100 nodes deployed in the network are still alive and maintain a 100% sensing coverage at the 1,500^th^ simulation round in the case I when using the ECHR protocol. By contrast, the networks using the other two protocols have almost stopped working when the simulation reached the 1,500^th^ round. Moreover, in case II, the average energy dissipation of the ECHR protocol is almost the same as the other two protocols before the 3,000^th^ round, but after that, the average energy dissipation of sensor nodes using the LEACH and the LEACH-Coverage-U protocols are both less than that of the ECHR protocol. This is because some sensor nodes in the networks using the former two protocols have run out of energy before 3,000^th^ round. In other words, the cluster heads of the LEACH and the LEACH-Coverage-U network only need to transmit the data collected by a small number of sensor nodes. Nevertheless, the sensor networks using the LEACH and LEACH-Coverage-U protocols have lost a 100% coverage ratio when the simulation reaches the 3000^th^ round. In this regard, the proposed ECHR protocol more efficiently utilizes the energy of the redundant nodes, so the network lifetime is prolonged while a full sensing coverage is retained.

Note that if the compression coefficient *μ* of Equation (12) is set at 0 which is the same as the settings in [4,18], the ECHR protocol provides 572.9% and 440.1% increases in service time with a 100% sensing coverage ratio comparing with the LEACH and LEACH-Coverage-U protocols in the case I. In the case II, the ECHR also increases 136.7% and 163.6% service time with a 100% sensing coverage ratio comparing with the LEACH and LEACH-Coverage-U protocols. These increases are better than the experimental results when the compression coefficient *μ* is set at 0.05.

**Figure 11.**
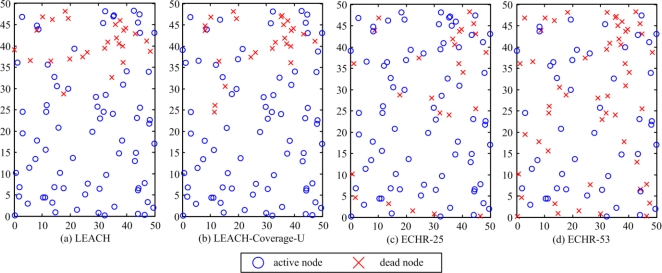
Distribution of alive and dead nodes yielded by (**a**) LEACH protocol, (**b**) LEACH-Coverage-U protocol, and (**c**) the proposed ECHR protocol. (**d**) Distribution of active and dead nodes before the network fails to maintain 100% sensing coverage obtained by applying the proposed ECHR protocol.

Figure 11 shows the distribution of active and dead nodes of the network in the case I. Figure 11(a) plots the distribution of the network topology of active and dead nodes when the number of dead nodes rises to 25, obtained by applying the LEACH protocol to the network.

In this case, most of the dead nodes are located at the upper side of the network, *i.e.*, distanced from the BS. This fact is because the energy consumption for transmitting data between the BS and these nodes are greater than the energy use when nodes are located at the bottom side. Figure 11(b) depicts the distribution of active and dead nodes when the number of dead nodes is equal to 25 by applying the LEACH-Coverage-U protocol to the network.

Comparing with Figure 11(a), the active nodes in Figure 11(b) are more dispersed because the locations of nodes are taken into account by the LEACH-Coverage-U protocol. However, the majority of dead nodes are still located at the upper side. Figure 11(c) shows the distribution of active and dead nodes when the number of dead node is equal to 25, obtained by applying the proposed ECHR protocol to the network. Because the ECHR protocol is able to manage the network with features of energy-balancing and coverage-preserving, the locations of dead nodes are more evenly distributed over the network, which can effectively prevent coverage holes from occurring. Furthermore, Figure 11(d) depicts the distributions of nodes before the network fails to maintain 100% sensing coverage. In this case, the dead nodes are equal to 53. The experiment results demonstrate that using the overlapped sensing ranges the proposed ECHR protocol utilizes the energy of the nodes more efficiently. Thus, the duration for maintaining full sensing coverage can be significantly prolonged.

## 6. Conclusions

In this paper a hierarchical routing algorithm, capable of energy-balancing and coverage-preservation designed for wireless sensor networks, is proposed. The proposed ECHR algorithm aims to prolong network lifetime with a full sensing coverage for mission-critical applications. Extending network lifetime without the risk of data loss is a basic QoS requirement in such applications. The main idea of the ECHR algorithm is that in the stage of root node selection both of the energy-balancing and coverage-preservation mechanisms are taken into account. With this root node selection scheme, the redundant nodes can be chosen as the root node in early stages. In order to enhance the performance of the ECHR algorithm, the distance and the residual energy of neighboring nodes is incorporated into the algorithm when choosing an energy-efficient route for each node. The simulation results show that the proposed ECHR algorithm is able to prolong the network lifetime while retaining a 100% coverage ratio in case I and case II. The proposed ECHR algorithm outperforms the existing routing protocols such as the LEACH and the LEACH-Coverage-U. These results suggest that the QoS-guaranteed coverage precedence for WSNs in mission critical applications could be achieved when using the ECHR protocol. Some further information of the network can be utilized to enhance the feasibility of function-specific algorithms in WSN-based mission-critical applications. For example, the link quality indication (LQI) and the received signal strength indication (RSSI) can be used to estimate the distance between nodes. Thereby, the proposed ECHR algorithm can be adopted without knowing the exact location of the sensor nodes. Such an issue is left to research.

## Figures and Tables

**Table 1.** Parameter settings used in the simulations.

**Parameter**	**Acronym**	Setting ^*^
Radio circuitry	*E_elec_*	50 nJ/bit
Transmit amplifier	*ɛ_amp_*	0.1 nJ/bit/m^γ^
Aggregation cost	*E_DA_*	5 nJ/bit
Data packet size	*k*	2,000 bits

* These parameter settings were adopted from [18].

**Table 2.** Comparison of network lifetimes under different sensing coverage ratios.

**Parameters**		**Coverage-time (Rounds)**			
***λ*_1_**	***λ*_2_**	**100%**	**95%**	**90%**	**80%**
0	3.3	1,094	1,334	1,359	1,381
0.3	3.3	1,583	1,675	1,692	1,709
0.7	3.3	1,590	1,677	1,692	1,711
10	3.3	1,578	1,657	1,671	1,687
50	3.3	1,581	1,652	1,671	1,687
0.7	0	1,580	1,653	1,672	1,688
0.7	1	1,585	1,666	1,682	1,697
0.7	3.3	1,590	1,677	1,692	1,711
0.7	10	1,584	1,677	1,693	1,709
0.7	50	1,585	1,672	1,690	1,707

## References

[1] Kuorilehto M, Hännikäinen M, Hämäläinen TD (2005). A Survey of Application Distribution in Wireless Sensor Networks. EURASIP J. Wirel. Commun. Netw.

[2] Brandi M, Grabner J, Kellner K, Seifert F, Nicolics J, Grabner S, Grabner G (2009). A Low-Cost Wireless Sensor System and Its Application in Dental Retainers. IEEE Sens. J.

[3] Stojmenović I (2005). Handbook of Sensor Networks: Algorithms and Architectures.

[4] Heinzelman WB, Chandraksaan AP, Balakrishnan H (2002). An Application-Specific Protocol Architecture for Wireless Sensor Networks. IEEE Tran. Wirel. Commun.

[5] Al-Karaki JN, Kamal AE (2004). Routing Techniques in Wireless Sensor Networks: A Survey. IEEE Wirel. Commun.

[6] Muruganathan SD, Fapojuwo AO A Hybrid Routing Protocol for Wireless Sensor Networks Based on a Two-Level Clustering Hierarchy with Enhanced Energy Efficiency.

[7] Diamond SM, Ceruti MG Application of Wireless Sensor Network to Military Information Integration.

[8] Fan GJ, Jin SY (2010). Coverage Problem in Wireless Sensor Network: A Survey. J. Netw.

[9] Lin JW, Chen YT (2008). Improving the Coverage of Randomized Scheduling in Wireless Sensor Networks. IEEE Trans. Wirel. Commun.

[10] Akkaya K, Younis M (2005). A Survey on Routing Protocols for Wireless Sensor Networks. Ad hoc Netw.

[11] Handy MJ, Haase M, Timmermann D Low Energy Clustering Hierarchy with Deterministic Cluster Head Selection.

[12] Younis O, Fahmy S (2004). HEED: A Hybrid, Energy-Efficient, Distributed Clustering Approach for ad hoc Sensor Network. IEEE Tran. Mob. Comput.

[13] Loh PKK, Jing HW, Pans Y (2009). Performance Evaluation of Efficient and Reliable Routing Protocols for Fixed-Power Sensor Networks. IEEE Trans. Wirel. Commun.

[14] Tan HO, Korpeoglu I, Stojmenovic I (2011). Computing Localized Power-Efficient Data Aggregation Trees for Sensor Networks. IEEE Trans. Parallel Distrib. Syst.

[15] Shu T, Krunz M, Vrudhula S Power Balanced Coverage-Time Optimization for Clustered Wireless Sensor Networks.

[16] Li C, Ye M, Chen G, Wu J An Energy-Efficient Unequal Clustering Mechanism for Wireless Sensor Networks.

[17] Lindsey S, Raghavendra C, Sivalingam KM (2002). Data Gathering Algorithms in Sensor Networks Using Energy Metrics. IEEE Trans. Parallel Distrib. Syst.

[18] Tsai YR (2007). Coverage-Preserving Routing Protocols for Randomly Distributed Wireless Sensor Networks. IEEE Trans. Wirel. Commun.

[19] Kumar N, Kumar M, Patel RB (2010). Coverage and Connectivity Aware Neural Network Based Energy Efficient Routing in Wireless Sensor Networks. Int. J. Appl. Graph Theory Wirel. ad hoc Netw. Sens. Netw.

[20] Noh Y, Lee S, Kim K Basestation-Aided Coverage-Aware Energy-Efficient Routing Protocol for Wireless Sensor Networks.

[21] Soro S, Heinzelman WB (2009). Cluster Head Election Techniques for Coverage Preservation in Wireless Sensor Networks. Ad Hoc Netw.

[22] Wang B, Lim HB, Ma D, Yang D A Coverage-Aware Clustering Protocol for Wireless Sensor Networks.

[23] Onur E, Ersoy C, Delic H, Akarun L (2007). Surveillance Wireless Sensor Networks: Deployment Quality Analysis. IEEE Netw.

[24] Jafari R, Encarnacao A, Zahoory A, Dabiri F, Noshadi H, Sarrafzadeh M Wireless Sensor Nnetworks for Health Monitoring.

[25] Rappaport TS (2002). Wireless Communications Principles and Practice.

[26] Cormen T, Leiserson C, Rivest R, Stein C (2003). Introduction to Algorithms.

